# Recent Developments in Sustainable Solubilization

**DOI:** 10.1002/cssc.202502326

**Published:** 2026-04-16

**Authors:** Eva Müller, Werner Kunz

**Affiliations:** ^1^ Solution chemistry group Institute of physical and theoretical chemistry Regensburg Germany

**Keywords:** green solubilization, sustainable solubilization, sustainable chemistry, sustainable solvents

## Abstract

The large‐scale use of toxic and environmentally hazardous solvents remains a major challenge in industrial manufacturing and consumer‐goods production. Conventional solubilization processes often depend on harsh conditions, including elevated temperatures and pressures, resulting in high energy consumption, health risks, and environmental pollution. Developing sustainable alternatives is therefore an urgent scientific and societal priority. This article discusses recent advances in green solubilization and emerging strategies aiming to reconcile efficiency with environmental compatibility. We address the future role of classical and “green” solvents, including ionic liquids (ILs) and natural deep eutectic solvents (NADES), and critically assess their benefits and limitations from a sustainability perspective. Particular emphasis is placed on water as the potentially “greenest” solvent, highlighting how its intrinsic tendency to form structured, heterogeneous environments can be advantageous or detrimental for solubilization. In this context, we examine mesoscale structuring, surfactant‐free microemulsions, and dynamic interfaces. Furthermore, naturally derived solubilizers such as hydrotropes, biosurfactants, and proteins are considered promising tools to enhance solubility while maintaining biocompatibility and low environmental impact. Selected examples from our own work illustrate how combining water‐based structuring with bio‐derived or benign additives can create new pathways toward energy‐efficient and sustainable solubilization technologies.

## Graphical abstract

We highlight several recent developments in sustainable solubilization. Particular attention is given to the use of bio‐based solvents derived from renewable resources, the strategic use of water as a solvent, and alternative, sustainable solubilizers such as hydrotropes. These approaches offer promising strategies for achieving more environmentally responsible and sustainable formulations.

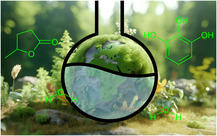

## 1 Introduction

Solvents like N‐methyl‐2‐pyrrolidon (NMP), dimethylsulfoxide (DMSO), or dimethylformamide (DMF) are still widely used, just like chlorinated solvents, etc., see Table 1. Of course, less toxic solvents also found their way to applications such as ethanol, and water is the most wide‐spread liquid. However, ethanol has a low ignition temperature (as does acetone, for example), and water has two other drawbacks. The first is evident and almost trivial: without additives, the solubility of organic substances in it is limited to not too hydrophobic ones. Second, it is often difficult to purify the used water. Distillation is very energy consuming and other techniques may not purify used water to a sufficient extent at a reasonable price and for big volumes.

**TABLE 1:** Annual production of some common solvents (tons).

Solvent	Annual production (2022/2023), tons	References
DMSO	41	[1, 2]
NMP	250	[3]
Chloroform	660	[4]
DMF	870	[5]
Ethyl acetate	4,530,000	[6]
Xylene	4,600,000	[7]
Acetone	7,730,000	[8]
Toluene	30,220,000	[9]
Ethanol	101,000,000	[10]
Methanol	108,700,000	[11]

So, there are two strategies to make more sustainable solutions: either finding alternative solvents or solvent mixtures or environmentally friendly additives to solubilize the desired reactants or products (or catalysts) in water, unless the subsequent separation of products from water is too difficult.

In what follows, we will give selected examples for innovations of the last 5 years for both pathways and specifically discuss in this context the future role of both classical and “green” solvents, including ionic liquids (ILs) and natural deep eutectic solvents (NADES). Briefly, we evaluate their advantages and limitations from a sustainability perspective. Particular attention is paid to water as the potentially “greenest” solvent. In this context, a central theme of this work is the development of sustainable strategies to enhance the solubility of organic compounds in water, a topic that has been comprehensively addressed in recent literature that even sets the methods in the context of economic and ecological benefit, considering the principles of green chemistry [12, 13]. Building on these established concepts, we integrate and extend the existing knowledge by incorporating recent advances, in particular those related to mesoscale structuring and dynamic solubilization phenomena. To this purpose, we highlight the inherent propensity of water to form structured, heterogeneous environments, which can act either as a barrier to solubilization or, when properly exploited, as a powerful means to enhance it. In this context, mesoscale structuring, surfactant‐free microemulsions (SFMEs), and highly dynamic interfaces are addressed as emerging concepts that bridge the gap between molecular solubility and phase‐separated systems. In addition, naturally derived solubilizers, including hydrotropes, biosurfactants, and proteins, are considered as promising tools to increase the aqueous solubility of organic compounds, while maintaining biocompatibility and a low environmental footprint. Selected examples from our own work illustrate how combining water‐based structuring with benign or bio‐derived additives can open new pathways toward energy‐efficient and sustainable solubilization technologies.

## 2 Different Strategies of Sustainable Solubilization

Moving away from conventional solvent systems and energy‐demanding solubilization techniques has become an important step toward greener chemistry. Instead of relying on hazardous substances or harsh operating conditions, a range of alternative approaches is now being explored. Among the most promising are the development of solvents with improved environmental profiles, the reconsideration of water as a versatile green medium, and the application of naturally derived solubilizers that open new pathways for sustainable formulation and processing.

### 2.1 Environmentally Friendly Solvents

In a very useful overview, the group of J.‐M. Aubry in France listed hundreds of solvents according to their toxicity [14]. Jessop et al. published an article about the most urgent need for new solvents [15], and there are numerous other interesting articles in this field, see, e.g [16, 17] and articles quoted herein. For more recent literature, see [16, 17, 18, 19, 20, 21, 22].

Of course, the best solvent would be no solvent. This can be the case if a reactant is liquid and the other reactants are soluble in it or if directly solid reactions can be performed, e.g., in ball mills. If this is not the case, ephemeral solvents could be considered, if possible. Examples are Jessop´s switchable solvents [23] or the in situ creation of a deep eutectic solvent (DES) with one of the reactants [24]. An interesting combination of the strategies of switchable solvents and DES is given in [25]. Here *Flos Chrysanthemi Indici* flower was extracted with a NADES of betaine and glycerol (1:2) combined with N, N‐dimethylcyclohexylamine and water (25:50:25 (v/v/v)). It was shown that the incorporation of the NADES accelerated the CO_2_‐triggered polarity switching by 32.7% and reduced the residual organic content in the aqueous phase by 73.7%. It further significantly enhanced extraction efficiencies by, e.g. increasing the total phenolics content from 43.5 to 71.4 mg g^−1^. However, in both cases, water is the main solvent (Table 2).

**TABLE 2:** Recent examples for applications of common solvents (switchable solvents, NADES, Ils, common organic solvents).

Solvent/ solvent system	Solvent type	Application	Findings	References
**Ionic liquids and NADES**				
[BMBIM]Br	Ionic liquid	Extraction of rutin from Pinus massaniana	Nearly double of yield with ionic liquid (41.07 mg g^−1^) compared to ethanol (22.5 mg g^−1^)	[26]
[BMIM][OAc] and [P_4_,_4_,_4_,_4_][OAc]	Ionic liquid	CO_2_ capture and catalystic activity in cycloadditions	ILs and PILs showing similar CO_2_ sorption with BMIM][OAc] and [P_4_, _4_,_4_,_4_][OAc] showing the highest capacities. Higher sorption does not directly correlate with better catalytic activity.	[26]
Fatty acid or N‐lauroyl amino acids + choline	Ionic liquid	Solubilization of barely water soluble drugs	Significant enhancement of curcumin and luteolin water solubility	[26]
Amino acid esters + drugs (e.g. salicylic acid, ibuprofen, methotrexate,...)	Ionic liquid	Solubilization of barely water soluble drugs	Significant enhancement of drug solubility while being biocompatible; increased bioavailability	[26]
Betain/ glycerol/ N, N‐dimethylcyclohexylamine/ water	NADES / switchable solvent	Extraction of polyphenols from Flos Chrysanthemi Indici	Incorporation of DES significantly reduced CO_2_ switching time by 32.7% and decreased the residual content in the aqueous phase by 73.7%	[25]
Cholin chloride/ citric acid/ water	NADES	Extraction of polyphenols from grape and olive pomace	Total phenolic content nearly doubled with the NADES (2190 mmol TE g^−1^ dw of pomace) in contrast to ethanol (1230 mmol TE g^−1^ dw of pomace) extraction	[27]
Cholin chloride/ glycerol	NADES	Extraction of proteins from soy	Significantly higher protein extraction yield (0.3462 g) as compared to sodium hydroxide extraction (0.3192 g)	[28]
Cholin chloride/ ethylene glycol	NADES	Polysaccharides from lotus leaves	5.38% of extraction yield and 82.10 of total polysaccharide content	[28]
Oleic acid/ thymol	NADES	Extraction of astaxanthin from Haematococcus puvialis	60% of astaxanthin recovered	[28]
Citric acid/1,2‐propanediol	NADES	Extraction of sideritis scardica	Slightly lower extraction yield than with 70% ethanol but extract much more biologically active	[29]
Choline chloride/ ethylene glycol or lactic acid or oxalic acid	NADES	Pretreatment of lignocellulosic biomass	The solubility of lignin in ChCl/Oxalic acid was the highest (47.85%) but the bagasse was carbonized after pretreatment. ChCl/Lactic acid was more suitable for cellulose separation while ChCl/Ethylene glycol, ChCl/Glycerol and ChCl/Urea had selectivity toward lignin separation.	[30]
Tetrabutylammonium bromide/ fatty acids/ melamine sponge	DES	Contaminant removal	Effective removal of about 70% of pesticides	[31]
Betaine hydrochloride/ ethylene glycol	NADES	Electrochemical applications	NADES are stable electrolytes allowing for high capacitance in supercapacitors	[32]
Choline chloride/ urea	NADES	Reaction medium for benzoxazines	Satisfactory yields of 81%–88%	[33]
**Classical solvents**				
Gammavalerolactone (GVL)	Solvent	Substitute for 2‐pyrrolidone and dimethylformamide	Applications in synthesis of certain polymers and pharmaceuticals, cleaning agent for varnishes, solubilizer in cosmetics and agroindustry	[34]
Gammavalerolactone	Solvent	Production of GVL	Advances in catalysts (e.g. zirconia based or zeolites) and thus elimination of hydrogen pressure and costly catalysts	[35]
Gammavalerolactone/ water	Solvent	Production of levulinic acid	Very high conversion yields of 76.1% with the ionic liquid catalyst [C_4_SO_3_Hmim][PhSO_3_]	[36]
Gammavalerolactone/ sulfuric acid/ aldehyde	Solvent	Cascade extraction of lignocellulosic biomass	Low extraction temperatures of 80°C and high lignin yields of 97%	[37, 38]
Gammavalerolactone	Solvent	Solvent for polyvinylchloride	Applicable considering its similarity to DMF and NMP; under current investigation	[34]
Gammavalerolactone	Solvent	Salting‐out liquid–liquid extraction for GVL recovery	With the help of sodium sulfate 99% recovery of GVL were achieved from an organosolv like mixture	[39]
Gammavalerolactone	Solvent	Solvent for photocatalytic reactions	GVL found to be C—H bond activator increasing reaction yield and kinetics in various reactions	[40]
Gammavalerolactone/ Lithium bis(trifluoromethanesulfonyl)imide	Solvent	Electrolyte for lithium ion batteries	Lower flammability risk, favorable conductivity, electrochemical stability and outstanding cycling stability.	[41]
2‐methyltetrahydrofuran (2‐MeTHF)	Solvent	Oil‐extraction of Actionostemma lobatum Maxim.kernel oil	30% higher oil yields than with n‐hexane. Highest tocopherol and carotene contents of all tested extraction media	[42]
2‐methyltetrahydrofuran	Solvent	Suzuki–Miyaura cross‐coupling between aryl halides and aromatic boronic acids	Very good yields and good alternative to classical toluene	[43]
2‐methyltetrahydrofuran	Solvent	Synthesis of antidiabetic triazolo‐pyridazine‐6.yl‐substituted	Yields of up to 98% at room temperature	[43]
Solketal and other glycerolderivatives	Solvent	Oleotrope for antioxidants for bio‐Diesel stabilization	Use of low toxic hydrophilic antioxidants possible. Dissolution of significantly higher amounts than with ethanol possible (e.g. 13% with ethanol and 17% of gallic acid with solketal)	[44]
Glycerol‐mono and dialkylethers	Solvent	Solvent for rarely soluble natural phenols	Significant solubility improvement for certain glycerol ethers (e.g. 75.7 mg/mL of caffeic acid in glycerol‐1‐methylether compared to 36.5 mg/mL in propylene glycol)	[45]
Triacetin/ ethanol	Solvent	Solvent mix for dissolution and extraction of curcumin	Significant solubility improvement up to 500% compared to pure ethanol	[46]

#### 2.1.1 Ionic Liquids

**FIGURE 1:**
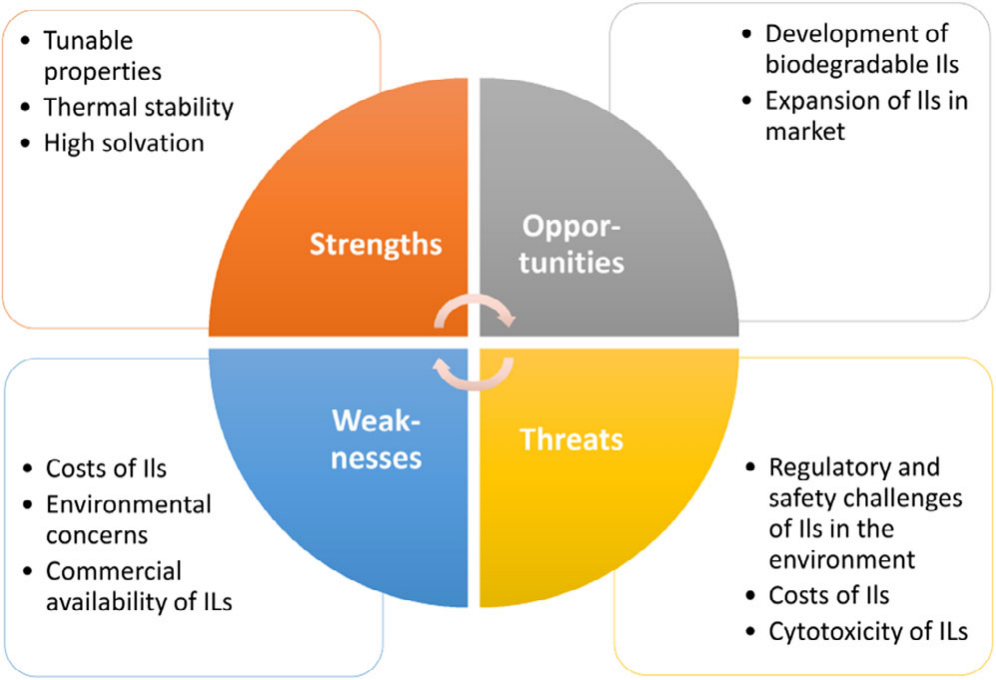
SWOT analysis presents the strength, weakness, opportunities, and threats relating to the use of ILs [48, 26].

Over quite a long time now, ILs have been considered as alternative “green” solvents [47, 48]. Of course, their very low vapor pressure (they are not volatile organic compounds (VOC)), their thermal stability, nonflammability, and adjustable polarity or viscosity make them attractive alternatives to conventional solvents. They have, e.g. emerged as versatile media for the extraction and isolation of natural products, offering tunable physicochemical properties derived from different cation–anion combinations, enabling high yields of compounds such as tannins, phenols, flavonoids, essential oils, alkaloids, carbohydrates, and lipids from plants, marine organisms, and microorganisms. Various IL‐assisted extraction methods —including ultrasonic‐ and microwave‐assisted protocols, enzyme‐assisted extraction, and aqueous biphasic systems—have demonstrated superior efficiency, particularly when process parameters such as solid‐to‐liquid ratio, time, temperature, and pH are optimized. E.g. the microwave‐assisted extraction of rutin with 70 vol% ethanol from Pinus massaniana yielded 22.5 mg g^−1^, whereas the IL [BMBIM]Br yielded 41.07 mg g^−1^ [26]. Third‐generation ILs, noted for their biocompatibility, cost‐effectiveness, and reduced environmental impact, are increasingly proposed for application in pharmaceutics due to their solubility, stability, and suitability for oral, topical, and transdermal drug delivery [49]. Switchable ILs responsive to external triggers (e.g. CO_2_) and polymeric ILs with enhanced properties expand their interdisciplinary potential [50]. For example, a recent study investigated ILs and poly(IL)s (PILs) as media for CO_2_ capture and conversion. The acetates [BMIM][OAc] and [P_4_,_4_,_4_,_4_][OAc] showed the highest CO_2_ solubility among the ILs, while bromide‐based PILs enhance CO_2_ uptake in water via cage‐like structuring. In CO_2_ cycloadditions, PIL catalysts and ILs were compared, revealing that DMSO suppresses the activity of ILs but enhances the performance of the catalyst. The results demonstrated that high CO_2_ sorption capacity of an IL does not necessarily translate into superior catalytic efficiency [50]. Recent reviews have highlighted their role in isolating bioactive compounds from medicinal plants. Microwave‐assisted systems, in particular, have been studied for their solvent–solute interactions and associated toxicity considerations [48]. Overall, ILs have proven effective for a wide profile of solubilization applications like the extracting of biomolecules, and the development of efficient recycling methods enhances their promise as cost‐effective and sustainable extractive solvents [48]. However, the most popular ones (mainly imidazolium‐ or alkylpyridinium‐based) turned out to be either nonbiodegradable, or at least slightly toxic (especially aquatoxic) or difficult to synthesize and to purify and thus quite expensive [48]. Although advances have been made in IL recovery and recycling, using strategies such as back extraction, distillation, salt‐induced phase separation, adsorption, or membrane separation, most of them still suffer from the difficulty to separate them from the solutes and to recover them easily [48]. Because of all these disadvantages, iILs found have only limited larger applications, often in niches and not so much for their claimed environmentally friendly properties (Figure 1).

The global market for ILs is projected to expand between 2025 and 2031 [51]. Their application potential continues to grow across a wide range of areas, including use as solvents, biocatalysts, operating fluids, in batteries, gas sorption, luminescence, light‐emitting and flexible devices, enzyme stabilization, pharmaceutical synthesis, and electrochemical or energy storage technologies [26, 47, 48, 49].

The versatility of ILs positions them as promising tools in both scientific research and industrial innovation, definitely making them relevant for tackling global sustainability challenges. Yet, future developments must particularly focus on designing biodegradable ILs and facilitating production and recycling processes.

#### 2.1.2 Deep Eutectic Solvents (DES) and Especially Natural Deep Eutectic Solvents (NADES)

Then, there is the class of DES, in some respects similar to ILs. Especially those that are made by mixing two natural components, so‐called NADES, attracted significant attention over the last few years [52]. Indeed, they have some advantages over ILs: they are easy to make, biodegradable and often of very low toxicity [53]. Despite these benefits, research activity on NADESs has lagged behind that on ILs, which have been studied since the early 1990s, whereas NADESs only gained wider attention after 2011 [53].

Particularly in natural product extraction, NADESs have shown strong potential as solubilizers and stabilizers, offering efficient recovery of valuable metabolites from medicinal plants, food by‐products, and agricultural residues. Studies have demonstrated enhanced solubility and stability of bioactive compounds such as polyphenols, leading to improved bioavailability without compromising biological activity [27]. E.g. a NADES of choline chloride and citric acid (2:1 + 30% water) was demonstrated to extract significantly higher amounts of polyphenols from food waste streams (grape and olive pomace) than ethanol [27]. Their application as extraction media for biological macromolecules like proteins, carbohydrates and lipids was likewise concluded to be generally superior to extractions with classical synthetic organic solvents [28]. Their low toxicity and biodegradability further support applications in pharmaceuticals, nutraceuticals, and cosmetics. For instance, Grozdanova et al. (2020) highlighted improvements in extraction efficiency and product safety when NADESs were used for plant metabolite isolation [29].

Beyond extraction, NADESs have been tested in biorefinery processes, where they can dissolve lignocellulosic biomass and thus provide a greener alternative to harsher solvents such as acids or alkalis [30]. Their potential extends into electrochemistry as sustainable electrolytes and into environmental applications such as pollutant removal from water and soil, where their tunable polarity and hydrophobicity allow selective extraction of contaminants [31, 32]. A recent review from Chevé‐Kools et al. highlights their potential, especially in the field of pharmaceutical applications, resuming examples for extraction and reaction media, as catalysts, and as active ingredients or solubilizing formulation additives [33]. Overall, NADESs represent a versatile, environmentally benign solvent platform with wide‐ranging potential applications from green chemistry to environmental remediation, although further studies are needed to optimize their design and broaden their practical implementation.

We expect some interesting future applications, inspired by their useful occurrence in nature, but until now, there utility seems still to be very restricted. Again, they are often difficult to separate from the solutes after use and also, just like in the case of ILs, a relatively high viscosity can be an issue.

In many published examples about the usefulness of ILs or DES, the authors do not compare their results with those that can be obtained with classical solvents such as ethanol. Further, a complete life cycle assessment (LCA) should be done so that a real comparison to classical solvents is possible. The perfectly “green” solvent will probably never exist, “green” always means “greener” than another substance.

#### 2.1.3 Classical Solvents

In the meantime, there is still a place for the search for more classical solvents. Here is an example: recently, we found that bio‐based gammavalerolactone (*ε*_*r*_ = 36.91; μ = 14.3 × 10^−30^ C m; $E_{T}^{N}$ = 0.301; b.p. = 207°C; m.p. = −31°C; GSK rating: yellow; no red‐flag H‐phrases) (GVL) [54], a solvent that is known since many decades, can be a suitable replacement for various more toxic and petrol‐based solvents such as DMF or NMP [34]. However, the application of GVL in large scales is still hampered by the small production scale (only some tons per year) and consequently its high price. This is about to change with the construction of a big production unit in Germany, which should be operational next year. GVL can be made from cellulose, sugars, or hemicellulose degradation products, such as furfural. If it was made from sugar, there is immediately be a discussion of the competition with food. However, one should keep in mind that there is an unhealthy overproduction of sugar, especially in Europe, where the plantation of sugar beets is still subsidized, despite the huge sugar production worldwide from sugar canes. Further advances are made in its synthetic route originating from levulinic acid and its esters, allowing for lower hydrogen pressure, while likewise costly metal catalysts may be eliminated [35]. It was even found to be able to promote the production of levulinic acid from bagasse as a cosolvent [36].

**FIGURE 2:**
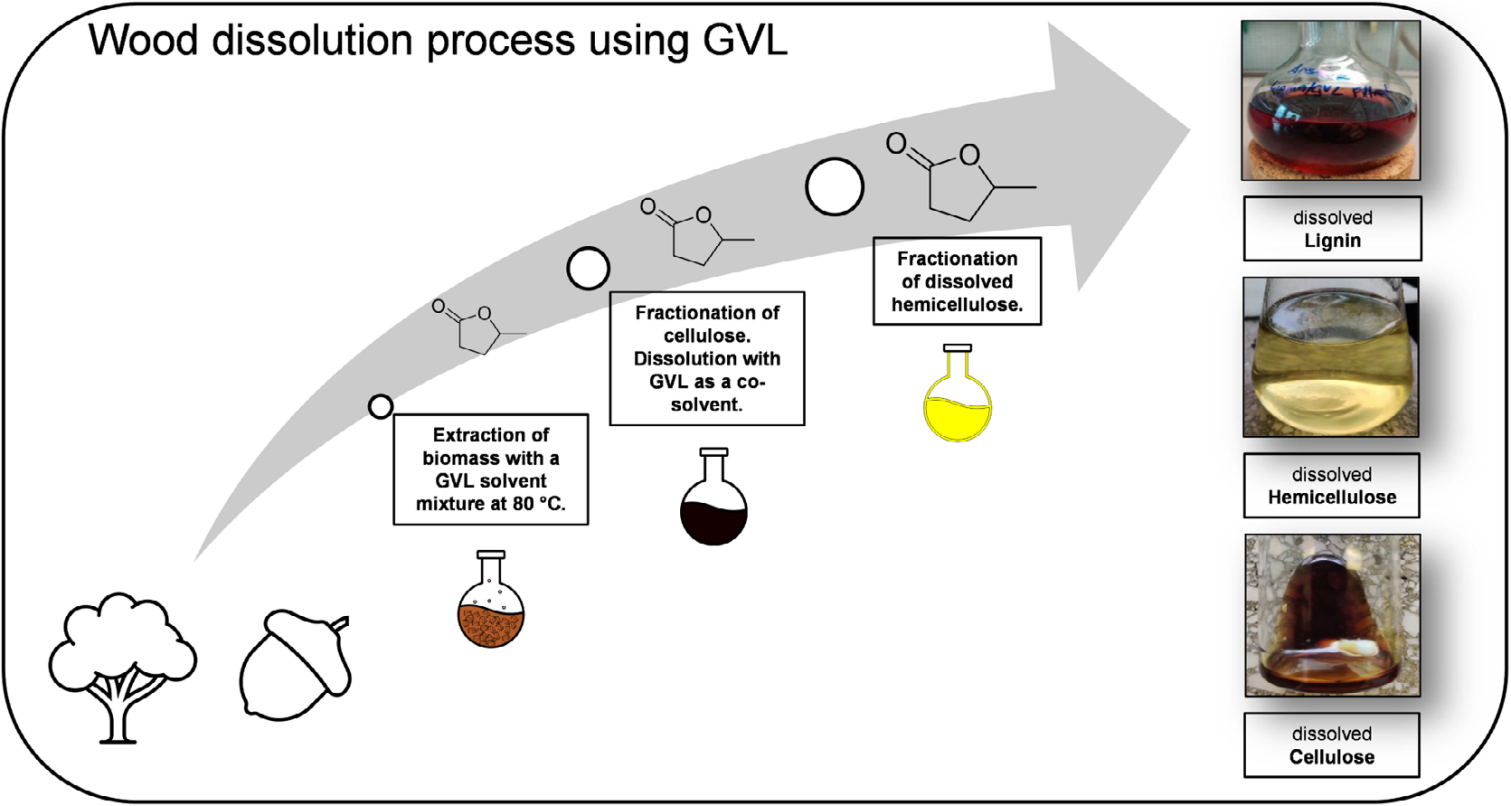
Schematic biomass dissolution process using GVL [37, 38].

As we found recently, GVL is even a suitable base solvent to dissolve entirely wood, with subsequent fractional precipitation of lignin, hemicellulose, and cellulose. For this purpose, it is sufficient to soak wood chips in GVL containing small amounts of sulfuric acid at a moderate temperature of 80°C. This process enables the dissolution of the major fractions of lignin and hemicellulose. By adding a small percentage of aldehyde, which helps prevent the recondensation of lignin subunits, the lignin yield can be increased to 97%. After diluting the extract with water, the lignin can be easily recovered, as it precipitates under these conditions. For further details on the dissolution process, see (Figure 2) [37, 38].

Once extracted and purified, lignin is a valuable raw material for aromatic compounds, as it mainly consists of the three alcohols: syringyl alcohol, guaiacyl alcohol, and p‐hydroxyphenyl alcohol, with their relative composition largely depending on the type of wood used. The use of lignin is expected to become increasingly important in the chemical industry's transition from petroleum‐based to bio‐based processes. Currently, the majority of lignin obtained from extraction processes (e.g., paper production) is still burned for energy, despite the wide range of potential synthetic routes to higher‐value materials. However, the only product currently synthesized from lignin is vanillin. Yet, 99% of artificial vanillin is still petroleum‐based [55].

After lignin is extracted from wood, the remaining cellulose loses its internal “glue” and becomes destabilized. Numerous publications discuss the dissolution of cellulose in various media. However, only the lyocell process has been industrially established as an alternative, less harmful technique compared to the carbon disulfide‐based viscose method [56].

While several wood‐dissolution processes have already been published in the recent years, they all leave one or another fundamental drawback, like high energy consumption and/or harmful or little environmentally benign chemicals like ammonia [37, 38]. The case of GVL shows that the clever choice and the implementation of bio‐based solvents allow for a major improvement of the sustainability of industrially important processes.

Note that GVL is miscible with water in all ratios and that it can even dissolve polymers such as PVC [34]. To remove GVL from solutions, it can be easily distilled at reasonable temperatures under vacuum, but it can also be salted out from water by the addition of appropriate salts like sodium chloride or sodium sulfate [39]. Numerous articles have been published in recent years about further promising applications of GVL. As particularly noteworthy potential applications, its supporting role in the photocatalytic activation of C—H bonds and its advantages in biomass pretreatment for subsequent refining [40, 57] and its application as a good solvent with high cycling capacity for electrical double layer capacitors can be mentioned [41].

Considering bio‐based solvents, 2‐methyltetrahydrofuran (*ε*_*r*_ = 6.97; μ = 4.6 × 10^−30^C m; $E_{T}^{N}$ = 0.179; b.p. = 78°C; m.p. = −136°C; GSK rating: Yellow; no red‐flag H‐phrases) (2‐MeTHF) turned out to be a very interesting alternative to hexane in the context of plant extraction [58]. In 2023, it was finally approved by the European Commission for this purpose [59]. Although not perfect, it is a valuable, greener alternative to toxic hexane [60]. Recently, a study showed nearly 30% higher oil yields in the extraction of Actionostemma lobatum Maxim.kernel oil. 2‐MeTHF is slightly more polar than hexane, so it will not extract exactly the same plant ingredients as hexane [42]. However, its boiling point is comparable, so it can be easily removed from the extracts by distillation. As a cyclic ether, there is a possible risk of explosion [61]. However, this risk can be minimized by the presence of a few percents of water [62]. Further, it was shown to be able to serve as “greener” alternative to conventional ether solvents in several organic reactions pertaining to organometallic‐, organo‐ and biocatalysis [43, 63].

Numerous further applications can be named for bio‐based solvents. Another very good example for sustainable solvent use is given by glycerol‐based solvents as oleotropes for hydrophilic natural antioxidants in biodiesel: further to making use of natural antioxidants, which are much less harmful than common crude oil based ones (like, e.g. butylated hydroxytoluene (BHT)), the utilized solubilizers, like solketal (b.p. ≈188°C–191°C; m.p. ≈−26°C; viscosity ≈11–12 mPa·s at 25°C), enhance fuel properties and are produced through simple addition reactions with glycerol, a common by‐product of industrial biodiesel production [44]. Likewise, the ether derivatives of glycerol, amongst which especially the short chain derivatives have shown comparatively low ecotoxicity, have also proven to be potent solubilizers for scarcely water‐soluble compounds, like coumaric, caffeic, or ferulic acid [45].

Advances are also made in the application of bio‐based solvents in organic synthesis. The already mentioned carbohydrate‐based ones GVL and 2‐methyltetrahydrofuran but also cyrene (b.p. ≈226°C; viscosity ≈14.5 cP at ~25°C) and dimethyl isosorbide (*ε*_*r*_ = 32; dipole moment: n.a; $E_{T}^{N}$ : n.a.; b.p  = 235°C; m.p. = −70°C; GSK rating: yellow; no red‐flag H‐phrases) just like terpene‐ and lignan‐based solvents like limonene and p‐cymene are of particular research interest in synthetic applications. Quadros et al. and Jordan et al. give a good overview of the recent developments [63, 64].

Using a single solvent exclusively is not the only way to create environmentally sustainable formulations. In many cases, combining two benign solvents can have significant synergistic effects. Shimizu and colleagues recently introduced a general theoretical framework to describe this phenomenon, known as the solubility isotherm theory [65]. The molecular interactions involved can differ considerably and are often difficult to elucidate unambiguously. For example, curcumin solubilization in a mixture of ethanol and triacetin—both of which are considered "drinkable" solvents—results in a bell‐shaped solubility curve, with maximum solubility being reached at around 40% ethanol and 60% triacetin and a solubility increase of 150% compared to pure triacetin and nearly 500% compared to pure ethanol [46]. The mechanism responsible for this enhanced solubilization remains unclear. One possible explanation is that triacetin disrupts the hydrogen‐bonding network of ethanol (the so‐called "living polymer"), thereby making ethanol molecules more available to solvate curcumin. However, this hypothesis still requires confirmation. In any case, such mixtures of two green solvents are worth closer attention.

In conclusion, we think that classical solvents will still have their place for industrial applications and that, until now, ILs and DES did not yet fulfill their promises concerning environmentally friendly alternatives to classical solvents.

### 2.2 Water as Potential “Green” Solvent

#### 2.2.1 Water as General Reaction Medium

Several research groups favor water as the potentially “greenest” solvent. Doubtlessly, water has its merits for reactions: for example, photocatalytic reactions are preferable in water, because the passage of radiation is less hindered than in other solvents [66]. Water can strongly accelerate organic and photochemical reactions compared to classical organic solvents, although limited substrate solubility often restricts its use. It has been shown that arenes, heteroarenes, enamines, or esters can form reactive aggregates in water due to melting‐point depression, creating oil–water interfaces in which hydrogen bonding to water activates the substrates and enhances photochemical reactivity. A striking example is the coupling of indole with isoquinoline‐1‐carbonitrile under 365 nm irradiation at 25°C, which proceeds in water with 92% yield, whereas the same reaction in methanol gives only 15% [66]. Additionally, many other types of reactions like conventional oxidations, reductions, additions, substitutions, condensations, cyclizations, or cross‐couplings were studied in water as a solvent [67, 68, 69]. Many organo‐catalyst reactions were shown to be effective for C—C bond‐forming reactions in water, notably aldol, Michael, and Mannich reactions. Chiral amines and proline derivatives catalyze aldol reactions to give β‐hydroxy carbonyl compounds with high enantioselectivity, while primary and secondary amines promote Michael additions between nucleophiles and Michael acceptors. Iminium‐ion‐based catalysts enable efficient Mannich reactions, affording β‐amino carbonyl products. For example, thiourea‐modified proline in the aldol reaction provides 99% yield with 99% ee, thiourea‐catalyzed Michael additions reach 97% yield and 99% ee, and proline‐mediated Mannich reactions give 95% yield with 93% ee [68]. Also, natural enzymes are made by nature to be effective in an aqueous environment, so these catalysts work ideally in aqueous solutions. As nature in general prefers an aqueous environment. Therefore, it is not astonishing and, in a sense, “biomimetic” that a paradigm shift has been claimed in literature: “Water as the reaction medium in organic chemistry: from our worst enemy to our best friend” is the title of a seminal article in *Chemical Science* from 2021 [69].

#### 2.2.2 Surfactants as Additives

**FIGURE 3:**
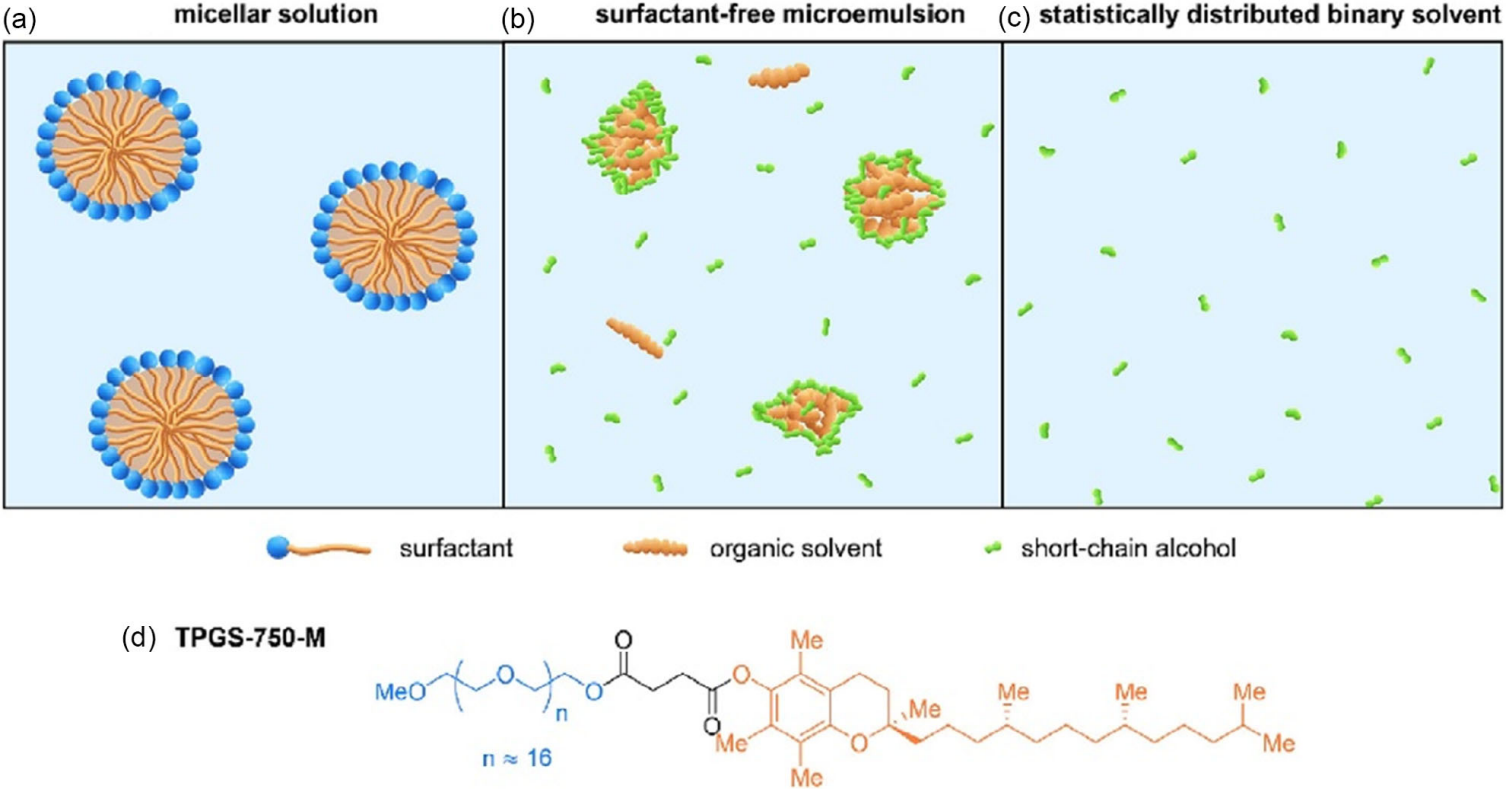
Schematic illustration of a micellar solution (a), a surfactant‐free microemulsion (b), and a statistically distributed binary solvent mixture (c). (d) Molecular structure of the surfactant TPGS‐750‐M. Figure taken from [70] with permission from Elsevier.

Of course, as already mentioned, the low water solubility of relevant organic reactants is the major drawback. To overcome this issue, usually surfactants are used to form micelles, in which the organic compounds are dissolved. It was recently shown that the ketoreductase ADH101 converts (E)‐4‐phenyl‐3‐buten‐2‐one in buffered water only up to about 57% after 1 h, at which point the reaction essentially levels off. In contrast, the addition of small amounts (2 wt%) of typical surfactants such as Tween 60, Triton X‐100, or TPGS‐750‐M (see Figure 3) to the same buffer markedly enhances both the reaction rate and the final conversion. A systematic increase in surfactant loading further raises the conversion. This behavior indicates that the micellar nanostructures formed in solution act as a reservoir for substrate and product, modulating their effective concentrations by shuttling them between the aqueous phase and the enzyme active site [69]. Even specially optimized surfactants have been proposed to make the solution less harmful and compatible with enzymes [69, 71]. However, surfactants have several shortcomings: they may be expensive, of some toxicity, difficult to remove, they can increase foaming, attach to surfaces, etc. So, the question is, to what extent are they really necessary? Over the last fewyears, we have conducted several studies concerning this question, and we could distinguish three cases:

In some cases, surfactants and the resulting micelle formation (above the critical micellar concentration) are really necessary. This is in particular the case when catalysts are required and when these catalysts must be fixed at a well‐formed interface between the organic reactants and some other reactants being dissolved preferentially in the outer aqueous phase around the micelles [69, 72, 73, 74]. Then, internal interfaces and a pronounced structuring of the aqueous solution are mandatory. To optimize these structures, a detailed knowledge of surfactants and—in the case of charged ones—of specific ion effects is helpful.

#### 2.2.3 Surfactant Free Microemulsions (SFMEs)

In other cases, still, an interface is preferable or even necessary, but this interface does not need to be as strongly developed as in the case of surfactant‐made micelles. Hydrotropes in water forming so‐called SFMEs may be sufficient [75]. The advantage is that by choosing uncharged hydrotropes such as short‐chain alcohols, they can be easily removed and recovered by simple distillation and they “only” form ill‐defined oily aggregates in the surrounding aqueous medium. We applied this concept, also called the “pre‐Ouzo” structuring, to control the size of polymer particles during their synthesis [76]. Here, the surfactant‐free swollen micelles functioned as a sort of nanocontainer just to limit the growth of the polymers. A surfactant‐free, mesostructured medium was developed for free‐radical polymerization of industrially relevant vinyl monomers, using water, methyl methacrylate, and simple alcohols (ethanol, n‐propanol, isopropanol, tert‐butanol) as hydrotropes. These SFMEs allow polymerization with either oil‐soluble thermal/photoinitiators or water‐soluble redox initiators. Except for ethanol, which forms a homogeneous solution, all alcohols generate mesostructured systems that strongly influence polymerization kinetics, molar mass, and morphology. Increasing alcohol content weakens mesostructuring and lowers both conversion and average molar mass, while ethanol yields particularly high molar masses. The results show that alcohol partitioning into the oil‐rich domains and the repulsive, alcohol‐rich interfacial regions control reactivity. Depending on the mesophase, polymer morphologies range from powder‐like to porous or dense transparent solids, placing SFME polymerization between classical solution and microemulsion/microsuspension processes [76]. An overview of potential and actual applications of SFMEs can be found in a recent review (Table 3) [77, 78].

**TABLE 3:** Recent examples of reactions considering medium‐compartmentalization or ‐structuring.

Reactants	Type	Reaction conditions	Findings	References
Indole + isoquinoline‐1‐carbonitrile	On‐water photoreaction	25°C, 365 nm, nitrogen, solvent	Reactant mix builds DES floating on water; water orients reactants due to H‐bonding; reaction yield in water 92% compared to, e.g. 15% in methanol; reaction kinetics significantly accelerated	[66]
(E)‐4‐phenyl‐3‐buten‐2‐one	Surfactant supported enzymatic reaction	37°C, TPGS‐750‐M (surfactant), water, ADH101, NaD^+^, NaDP^+^, i‐PrOH	Improved rate and level of conversion in presence of surfactant	[69]
(E)‐4‐phenyl‐3‐buten‐2‐one	Molecularly dissolved or SFME supported reaction	37°C, ADH101, NaD^+^, NaDP^+^, i‐PrOH, water (and benzylic alcohol)	Similar or even higher yields at lower temperatures in solvent mixtures than with surfactant	[70]
Methyl methacrylate	Polymerization in SFME	25°C, potassium persulfate and sodium sulfite, water, varying alcohols	Type of alcohol and structure of reaction medium have strong influence on morphology of the polymer. System can lead to comparable results to surfactant based microemulsions	[76]

#### 2.2.4 Comparison of Molecular Dissolution, Surfactants, and SFMEs

The third case concerns reactions that, to our surprise, do not need at all internal interphases, but “only” a sufficient cosolubility of the reactants. An increase in substrate solubility by the addition of a suitable cosolvent can, in many cases, be sufficient to optimize organic reactions in water, without the need for internal interphases such as those provided by micelles. In aqueous cosolvent mixtures, enhanced mutual solubility of the reactants facilitates effective molecular contact and thus promotes reactivity, a phenomenon that has been observed in numerous organic transformations [69]. We verified this concept by revisiting a representative tandem one‐pot reaction consisting of a Pd‐catalyzed Heck coupling followed by an enzyme‐catalyzed reduction. In contrast to earlier reports claiming that surfactants and micellar structuring are essential [79], we demonstrated that comparable or even improved yields can be achieved in the complete absence of surfactants, simply by adding an appropriate amount of *i*‐propanol to ensure sufficient solubility of all reaction partners. The solvent composition and structuring strongly influence both Pd‐catalyzed Heck couplings and ADH‐catalyzed ketone reductions. Micellar TPGS‐750‐M solutions were compared with surfactant‐free water/isopropanol (IPA) mixtures and with ternary water/IPA/benzyl alcohol (BA) systems of different mesostructure. For the ADH reaction, no micellar protection is required; rather, IPA induces a salting‐out effect that preserves enzyme folding and activity, giving 70%–80% yield in both unstructured and structured mixtures, and up to ~100% in weakly structured water/IPA/BA. Similarly, Heck reactions in these surfactant‐free media reach 75%–100% yield and even allow lowering the temperature from 45°C to 25°C, with up to 96% yield in the SFME‐system. As a consequence, one‐pot coupling of Heck and enzymatic reduction is possible without surfactants, demonstrating that sufficient solubility and stability of all components, rather than micellar nanoreactors, are the decisive factors [70]. Most water‐based catalysis studies primarily focus on enabling reactions in water using surfactants, while direct experimental comparisons to cosolvent‐only conditions remain rare. Where such comparisons are discussed, they typically appear in broader reviews or mechanistic contexts rather than as focused experimental benchmarks, highlighting that concrete head‐to‐head examples, like ours, are still missing.

### 2.3 Hydrotropy and Especially Natural Solubilizers as a Lever for Sustainable Solubilization

To make water a solubilization or reaction medium for scarcely hydrophilic compounds, usually additives are needed, as discussed in the preceding section.

A sustainable solubilization strategy must consider not only the efficacy in promoting reactions or extractions, but also the environmental and socioeconomic implications of the additive itself, including source, production/extraction, required dosage, biodegradability, and postuse management. Biosurfactants—amphiphilic molecules produced by microorganisms—offer notable sustainability advantages, such as biodegradability and low toxicity, and can be obtained from feedstocks like agricultural residues or waste streams [80].

Well‐known classes of biosurfactants include glycolipids such as rhamnolipids (produced mainly by *Pseudomonas* spp. from, e.g. olive oil or soybean oil refinery waste) and sophorolipids (produced by yeasts such as *Starmerella bombicola* *from*, e.g. canola oil or animal fat), as well as lipopeptides like surfactin (produced by, e.g. *Bacillus subtilis* *from*, e.g. sucrose or vegetable oil) [80]. Glycolipid‐producing microorganisms can achieve substantial yields under optimized fermentation. For instance, *Pseudomonas aeruginosa* cultivated on sunflower seed shells was reported to produce up to ~10.2 g L^−1^ rhamnolipids under optimized conditions [81]. Moreover, sophorolipid biosurfactants have been produced at industrially relevant scales, with optimized processes yielding over 55 g L^−1^ in bioreactor systems [82]. Several industrial sites and companies are already engaged in the production of biosurfactants, with current production capacities at individual facilities of the order of about 1000 t yr^−1^ and planned expansions to approximately 15,000 t yr^−1^ in the coming years [83]. Yet, because biosurfactant formation is a microbially driven process, operational parameters such as stirring intensity, pH, metal ion availability, temperature, aeration, dilution rate, and the nature of the carbon and nitrogen sources strongly influence the yield, composition, and functionality of the product. Modifying these cultivation conditions can even lead a single microbial strain to synthesize different types of biosurfactants, for instance, when alternative substrates are supplied. Therefore, a systematic investigation of these factors allows targeted tuning and optimization of the physicochemical properties of the biosurfactant [80].

Studies have shown that certain biosurfactants, such as rhamnolipids, are inherently biodegradable and exhibit relatively low acute toxicity. In controlled respirometry tests according to OECD guidelines, rhamnolipids demonstrated biodegradation rates of 34%–92% mineralization, indicating good environmental degradability, and acute toxicity (Microtox 5 min EC_50_) values ranging from 39.6 to 87.5 µM, classifying them as slightly toxic under US EPA categories [84].

Biosurfactants are used across sectors, including household and personal care products, bioremediation, agriculture, enhanced oil recovery, detergents and pharmaceutical delivery, owing to their biodegradability, low toxicity and surface/interfacial activity. In many applications, effective concentrations range broadly but often lie in the sub‐percent to few percent weight/volume domain; for example rhamnolipid biosurfactants exhibit significant interfacial and solubilization activity at concentrations below 1% (w/v), as reflected in their low critical micelle concentrations and phase‐behavior properties in environmental applications., whereas sophorolipids can lower water surface tension from ~72 to ~27 mN m^−1^ at similar ranges [80]. Yet, biosurfactants are particularly attractive for environmental and agricultural applications, where they can outperform conventional synthetic surfactants due to their biodegradability, high surface activity, low toxicity, structural specificity, and stability over a wide range of pH, salinity, and temperature. These properties make them especially suitable for sustainable remediation and crop protection, as they combine high efficiency with low ecological impact. Their use has been demonstrated in diverse fields such as heavy‐metal removal from soils and waters, enhancement of phyto‐ and bioremediation, reclamation of hydrocarbon‐contaminated sites, improvement of pesticide and biopesticide formulations, and micronutrient delivery to plants. For example, the lipopeptide biosurfactants produced by *Bacillus* sp. MSI 54 were reported to remove 75.5% Hg, 97.73% Pb, 89.5% Mn, and 99.93% Cd from aqueous solutions containing 1000 ppm of the respective metals. Similarly, a biosurfactant from *Bacillus cereus* NWUAB01 achieved removal efficiencies of 69% for Pb, 54% for Cd, and 43% for Cr from contaminated soil. Beyond remediation, biosurfactants also display strong biocontrol activity: *Bacillus subtilis* AKP produces surfactin, iturin, and fengycin homologs that inhibit the chili anthracnose pathogen *Colletotrichum capsici* by 61.5% in dual‐culture assays (Kumar et al., 2021). Likewise, insecticidal and larvicidal effects have been observed, for instance, for lipopeptides from *Bacillus velezensis* PHP1601 against the blowfly *Lucilia cuprina* and for *Bacillus amyloliquefaciens* AG1 against the aphid *Myzus persicae* [85].

Downstream processing for recovery of biosurfactants frequently involves foam fractionation, solvent extraction, membrane separation or precipitation, with overall recovery efficiencies typically lower than those of synthetic surfactants due to structural complexity and microbial metabolite mixtures [86].

However, production costs and yields remain important constraints: techno‐economic analysis of surfactin production has indicated that low fermentation yields and high processing costs necessitate relatively high minimum selling prices for economic viability. In a conceptual design study, minimum surfactin selling prices in the range of 29–31 USD kg^−1^ were required for a process to break even, underscoring the current cost and scalability challenges of biosurfactant production [87]. Such characteristics must be weighed against benefits like renewable feedstock use and enhanced biodegradability.

On the other hand, apart from biosurfactants, hydrotropes are an important class to be mentioned for the sustainable solubilization of solutes in water [88]. Especially, natural hydrotropes must be mentioned. Classical ones amongst them—small organic solubilizing agents such as sodium benzoate, sodium salicylate or urea—can increase aqueous solubility of poorly soluble compounds by up to two orders of magnitude without requiring micelle formation [88]. For example, the solubility of the drug Aceclofenac can be increased 400‐fold in an aqueous 2.5 M sodium salicylate solution, while even a factor of 1000 was reported in a 2 M sodium benzoate solution. The achieved solubility increase of the drug Diacerein in an 8 M aqueous solution of urea was reported to be 270‐fold [88]. However, such hydrotropic effects are achieved at relatively high additive concentrations, but avoid the complexities of biosurfactant fermentation and purification.

Yet, there is still important ongoing research to find hydrotropic substances that can effectively fulfill the role of solubilizers contributing to sustainable solubilization.

E.g. hydrotrope‐assisted cosolvent systems have recently been shown also to play a decisive role in energy‐relevant aqueous media. In nanoengineered Zn‐ion electrolytes, the combination of a hydrophobic hydrofluoroether cosolvent with fluorinated hydrotrope molecules enables the formation of a hydrophilic–hydrophobic solvation sheath that effectively lowers the activity of water and suppresses its electrochemical decomposition. By confining water molecules through hydrogen bonding in an inner hydrophilic layer and repulsion by an outer hydrophobic layer, an average Zn plating/stripping reversibility of 99.92% over more than 4000 cycles was achieved at 2.0 mA cm^−2^ and 2.0 mAh cm^−2^ in Zn||Cu cells. Remarkably, the resulting aqueous–hydrotrope hybrid electrolyte allowed stable and highly reversible operation of Zn||VOPO_4_·2H_2_O cells over an exceptionally wide temperature window from −80°C to +60°C [89].

The preceding example illustrates that hydrotropic effects can also play an important role in future energy‐storage media; however, the hydrotropes employed in this case are themselves of limited sustainability. In contrast, we recently discovered that natural antioxidants can also play the role of solubilization enhancers and consequently can have the double role of chemical stabilizers (against oxidation) and hydrotropes [90, 91]. In this context, a panel of plant‐based hormone and phenolic compounds was tested for their solubilizing properties. It was found that strong hydrotrope‐solute interactions can even stabilize certain compounds, such as riboflavin, against oxidative stress.

Several important natural phenols, like ferulic acid, bear a carboxylic group. If this group is protonated (at low pH below about 4.5), they are scarcely water soluble. However, at neutral to moderately basic pH, these polyphenols are deprotonated and as a consequence reasonably soluble, without losing their antioxidative power. But additionally, we found that some of these potent antioxidants can also play the role of a solubilizer. We could, e.g. show that the vitamin riboflavin was 15 times more effectively solubilized by selected natural antioxidative phenolates than with sodium dodecylsulphate or even 75 times more than the classically applied nicotinamide [91, 92]. The solubilization mechanism can be different and also depends on the type of solute. In some cases, the structure of the phenol makes it suitable as a classical hydrotrope. In other cases, they may break stacks within aggregates of the solutes. A further, or coupled, mechanism may be the formation of strong complexes between the charged and water‐soluble polyphenol and the solute to be solubilized [92]. This concept of solubilization enhancement is already demonstrated by nature. Biologically active compounds, such as polyphenols with strong antioxidant properties or alkaloids, often exhibit poor solubility due to stacking interactions. In plants, these compounds are typically paired with well‐water‐soluble counterparts, likely to enhance the bioavailability of the active ingredients through solubilization. The complex of caffeine with the soluble chlorogenic acid in coffee exemplifies this natural solubilization strategy. This dual role of additives allows for a general reduction in the number of functional compounds needed in a mixture. The basic principle of using less also helps minimize potential negative environmental impacts.

In the realm of hydrotropic solubilizers, the relatively old but not widely applied concept of facilitated hydrotropy fundamentally contributes to the reduction of required chemical compounds [88, 93]. This strategy involves finding a hydrotrope that is an excellent solubilizer for the solute, but on its own poorly soluble in the solvent. By adding a small amount of a solubilizer for this hydrotrope, a significant synergistic effect is achieved [88]. The remarkable efficiency of this approach was recently demonstrated in the solubilization of quercetin, which has poor water solubility of only 0.012 mmol L^−1^ primarily due to pi‐stacking interactions. Using a combination of pyrogallol and phloroglucinol, the solubility of quercetin was significantly enhanced. Pyrogallol, known for its good water solubility, is a well‐established hydrotrope. Phloroglucinol, although poorly water‐soluble, showed comparatively better solubilization performance for the polyphenol. While a 0.1 M solution of pyrogallol in water led to a solubility of quercetin of 0.025 mmol L^−1^, phloroglucinol increased the solubility to 0.032 mmol L^−1^. The combination of the two solubilizers proved to have a significant synergistic effect. Experiments of 1 and 10 wt% of pyrogallol, which were saturated with phloroglucinol, lead to further solubility boosts of 61% and an additional 38% of quercetin, respectively, which would not be explicable by mere additivity considering the low solubility of pure phloroglucinol. Phloroglucinol, being very planar with substituents that allow close approximation to similarly planar compounds like quercetin, acts as an efficient stacking‐breaking agent. On the other hand, pyrogallol increases the overall solubility of phloroglucinol in water due to its planarity and the ability to form hydrogen bonds with phloroglucinol [94].

**FIGURE 4:**
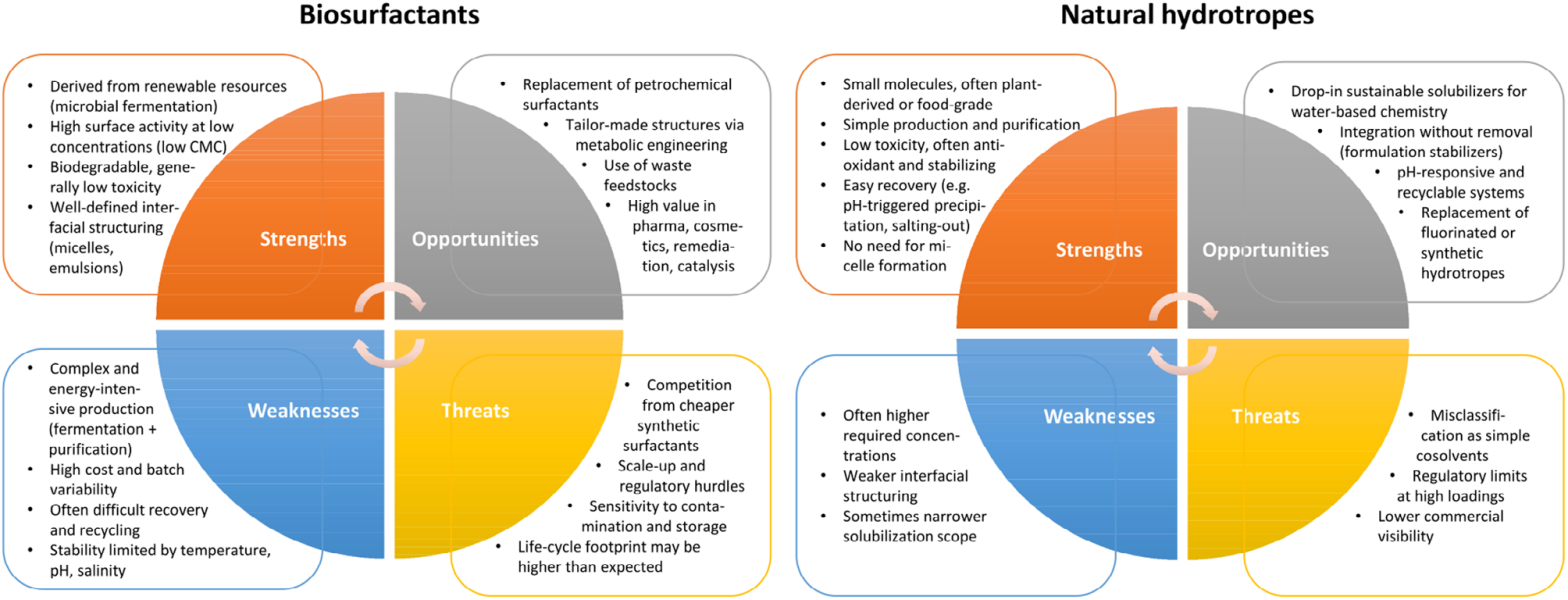
SWOT analysis presents the strength, weakness, opportunities and threats relating to the use of biosurfactants and natural hydrotropes [80, 82, 88].

Hydrotropes or natural hydrotropes can, in some cases, be disadvantaged compared to biosurfactants or surfactants in general, especially with respect to the solute‐to‐solubilizer ratio, although this is not necessarily the case when an appropriate hydrotrope, with more specific interactions with the solute, like H‐bonds or π‐stacking, is selected. In particular, phenolate‐based hydrotropes can offer additional advantages. Although, depending on the type of hydrotrope, they may be recovered by, e.g. pH adjustment followed by precipitation, enabling straightforward separation and reuse. An especially elegant and sustainable aspect of the described strategy is that many phenolic hydrotropes exhibit intrinsic antioxidant activity and low toxicity, allowing them to remain in the final formulation, while even improving their stability [91], in numerous applications. Beyond solubilization, their stabilizing properties can thus be exploited without requiring removal, contributing to both process simplification and improved overall sustainability. A general SWOT analysis for biosurfactants and natural hydrotropes can be found in Figure 4. However, the partially contrasting profiles of biosurfactants and natural hydrotropes suggest that a comparative sustainability assessment—including feedstock renewability, production/extraction effort, additive amount, biodegradability, and potential for recovery or recycling—is necessary for the specific application case to select optimal solubilization strategies for sustainable chemical processes.

Yet, sustainable solubilization also encloses the use of other natural compounds, further to classical hydrotropes or antioxidants. This strategy is particularly elegant, as important sustainability parameters like biodegradation have already been addressed and solved by nature.

Amongst these, proteins shall be highlighted due to their easy and comparatively cheap availability. Proteins are multifunctional biopolymers. The molecular structures are various and so are the possibilities to solubilize compounds of interest. Micelles, complexes, capsules, gels, and many more nanoarchitectures can be built up via different interaction mechanisms, like self‐assembly of proteins, hydrophobic or electrostatic interactions, and hydrogen bonding between the solute and the binding sites of the proteins [95].

An important application of proteins as solubilizers can be found in the food industry. Already for a while, it has become of great interest to design so‐called functional foods. The term describes food products that are enriched in nutraceuticals like antioxidants and vitamins. Many of those bioactive compounds cannot be added in pure form due to their low aqueous solubility or low stability. Proteins as solubility‐enhancing agents, derived from animals or plants, are of great interest as they are already part of the daily diet and thus, biocompatible [96, 97].

We recently found that curcumin, an important nutrient, which is a polyphenol with low water solubility and low stability, cannot only be solubilized but also be significantly stabilized in aqueous commercial pea protein isolate solution. In this study, pea protein isolate nanoparticles were generated by a pH‐triggered aggregation process and used to study the uptake and release of the polyphenol curcumin. Immediately after preparation, a pronounced supersaturation of curcumin was detected. Upon continuous stirring for 24 h, however, 96% of the initially dissolved curcumin reprecipitated, leaving a stable dispersion of pea‐protein–curcumin aggregates (PPICur). The corresponding curcumin concentrations decreased from 1.39 ± 0.093 mg mL^−1^ directly after preparation to 0.0528 ± 0.0088 mg mL^−1^ after 24 h. Considering literature data, in which curcumin solubility in neutral water or buffer is only 11–10 µg mL^−1^ and rapid degradation occurs within minutes without stabilization, these values demonstrate a strong solubilizing and protective effect of the protein matrix. UV–vis analysis of ethanolic extracts confirmed that curcumin initially adsorbs to the surface of the protein aggregates, from where a large fraction desorbs and precipitates, while a smaller but stable amount remains bound and protected by the pea protein nanoparticles. Further details can be found in [98].

Note that in nature, it can be important to keep proteins dissolved in water and to prevent proteins from aggregation and fibrillation. In this context, it was discovered that ATP can (and probably does) play the role of a “biological” hydrotrope or solubilizer [99]. It is particularly interesting that ATP is “salting‐out” for hydrophobic substances, but “salting‐in” for relevant proteins. A closer analysis, however, showed that these effects do not arise from classical hydrotropy. The prevention of aggregation is mainly governed by a specific ion effect of the highly charged, strongly hydrated ATP anion within the Hofmeister series, whereas inhibition of fibrillation is dominated by π–π interactions between the adenosine moiety and aromatic amino acid residues. In contrast, ATP does not increase the solubility of hydrophobic organic molecules; rather, it reduces it, demonstrating that ATP does not behave as a conventional hydrotrope. For details and alternatives to ATP, see [100]. This effect is not specific to ATP alone but can be generalized to a broader range of hydrotropic solubilizers with charged headgroups. Charged amphiphilic molecules, like hydrotropes, enhance the solubility of hydrophobic compounds in water but also exhibit specific ion effects. These effects can either enhance or reduce the solubilizing power, depending on ion hydration. In some cases, they may even turn a solubilizer into a salting‐out agent, or “antihydrotrope”. The delicate balance between Hofmeister effects from ionic headgroups and the hydrophobicity of the molecule, considering factors like molecular size and electron‐withdrawing groups, is decisive (Table 4) [101].

**TABLE 4:** Recent examples for interesting applications of natural solubilizers.

Solute / active	Natural solubilizer	Application	Findings	References
**Biosurfactants**				
Heavy metals	Lipopeptide from Bacillus cereus NWUAB01	Heavy metal removal from soil	Pb (69%), Cd (54%), and Cr (43%)	[85]
Fungus	Lipopeptide from Bacillus subtilis	Antifungal properties	61.5% inhibition of fungal growth	[85]
Lucilia cuprina larves	Lipopeptide from Bacillus subtilis Bacillus velezensis	Insecticidal properties	Antagonistic properties against the larval phase of Lucilia cuprina (Diptera: Calliphoridae), a blowfly pest of notable agricultural importance	[85]
**Natural hydrotropes**				
Aceclofenac	Sodium salicylate 2.5 M	Drug dissolution in water	400‐fold solubility increase	[88]
Aceclofenac	Sodium benzoate 2 M	Drug dissolution in water	1000‐fold solubility increase	[88]
Diacerein	Urea 8 M	Drug dissolution in water	270‐fold solubility increase	[88]
Amyloid fibrilles	Adenosinetriphosphate	Protein dissolution	Strong salting‐in of proteins, while hydrophobic dyes are salted out	[100]
Riboflavin	Plant‐based phenolic antioxidants	Vitamin dissolution	Solute 15‐fold more effectively solubilized than with sodium dodecylsulphate or 75‐fold than nicotineamide	[91, 92]
Quercetin	Pyrogallol + phloroglucinol	Drug dissolution in water	Synergistic hydrotropic effect with a solubility increase of 61%	[94]
**Proteins**				
Curcumin	Pea protein isolate	Drug dissolution in water	Approximately 50‐fold increase of curcumin solubility and stabilization against degradation	[98]

However, the phase separation of proteins from their aqueous environment can create a second, hydrophobic phase in thermodynamic equilibrium with the aqueous phase, which resembles the SFMEs discussed before. Nanodroplets or microdroplets of the organic protein phase are surrounded by the aqueous phase without a defined interfacial amphiphilic film. As such, material exchange is not hindered. It seems that this is a very common feature and is present in nature [102]. It is a second type of spontaneous phase equilibrium, further to the presence of organic organelles, like ribosomes, which are surrounded by amphiphilic membranes and therefore are similar to surfactant‐containing, classical microemulsions or emulsions.

## 3 Summary and Outlook

Solvents are still too often regarded as unavoidable auxiliaries whose selection is of secondary importance compared to catalysts, reagents, or reaction design. The developments discussed throughout this work clearly demonstrate that this view is no longer tenable. With growing environmental awareness and increasingly stringent regulations, not only the volatility and toxicity of solvents but also their origin, production routes, recyclability, and ultimate fate in the environment must be considered as integral parts of process design. In this context, solvent choice becomes a decisive lever for improving the overall sustainability, efficiency, and robustness of chemical transformations and formulations.

Water will undoubtedly play a central role in this transition. Beyond its obvious advantages in terms of availability, nonflammability, and low toxicity, it has been shown that water is far from being an inert background medium. Hydrogen bonding, hydrophobic effects, aggregation phenomena, and interfacial structuring can profoundly influence reactivity, selectivity, and even reaction mechanisms. The examples discussed—ranging from organo‐catalysis and photocatalysis to transition‐metal catalysis and biocatalytic cascade reactions—illustrate that high efficiencies can be achieved in aqueous systems, sometimes surpassing those obtained in classical organic solvents. Importantly, it has also become clear that internal interfaces such as micelles are not always required; in many cases, a sufficient cosolubility provided by carefully chosen cosolvents or hydrotropes is enough to ensure high activity and stability, while avoiding the additional complexity and downstream separation issues associated with surfactants.

At the same time, alternative molecular solvents such as gammavalerolactone, solketal, and 3‐methyl‐THF demonstrate that the palette of environmentally more acceptable organic media is steadily expanding. These solvents, derived from renewable feedstocks and offering favorable toxicological and physicochemical profiles, represent realistic drop‐in replacements for problematic dipolar aprotic solvents such as NMP or DMF. In contrast, despite their conceptual appeal and tunability, ILs and DES have so far not fulfilled early expectations for broad industrial deployment. Issues related to viscosity, cost, purification, recyclability, long‐term toxicity, and life‐cycle assessment continue to limit their large‐scale use, even though niche applications, for example, in extraction, catalysis, or electrochemistry, are emerging.

Finally, concepts such as hydrotropy, switchable solvents, and solvent systems capable of dynamic structuring underline that future solvent design will likely move beyond the simple dichotomy of “water versus organic solvent”. Instead, adaptive, multifunctional media that combine solubilization, stabilization, and sometimes even catalytic or protective functions will become increasingly important. All these insights show that solvents are not merely passive components but active elements of reaction systems whose intelligent selection and design can decisively enhance sustainability and performance. Thus, although the topic of solvents is as old as chemistry itself, it remains far from old‐fashioned and still offers substantial room for innovation.

## Author Contributions

**Eva Müller**: validation (equal), visualization (lead), writing – original draft (equal), writing – review & editing (lead). **Werner Kunz**: validation (equal), writing – original draft (equal), writing review & editing (supporting).

## Conflicts of Interest

The authors declare no conflicts of interest.
